# To Use or Not to Use Metformin in Cerebral Ischemia: A Review of the Application of Metformin in Stroke Rodents

**DOI:** 10.1155/2017/9756429

**Published:** 2017-05-28

**Authors:** Isaac Arbeláez-Quintero, Mauricio Palacios

**Affiliations:** Centro de Estudios Cerebrales, Facultad de Salud, Universidad del Valle, Cali, Colombia

## Abstract

Ischemic strokes are major causes of death and disability. Searching for potential therapeutic strategies to prevent and treat stroke is necessary, given the increase in overall life expectancy. Epidemiological reports indicate that metformin is an oral antidiabetic medication that can reduce the incidence of ischemic events in patients with diabetes* mellitus*. Its mechanism of action has not been elucidated, but metformin pleiotropic effects involve actions in addition to glycemic control. AMPK activation has been described as one of the pharmacological mechanisms that explain the action of metformin and that lead to neuroprotective effects. Most experiments done in the cerebral ischemia model, via middle cerebral artery occlusion in rodents (MCAO), had positive results favoring metformin's neuroprotective role and involve several cellular pathways like oxidative stress, endothelial nitric oxide synthase activation, activation of angiogenesis and neurogenesis, autophagia, and apoptosis. We will review the pharmacological properties of metformin and its possible mechanisms that lead to neuroprotection in cerebral ischemia.

## 1. Introduction

Metformin is an antidiabetic drug from the dimethyl-biguanide family. It was synthesized in 1920. It originates from the European* Galega officinalis* plant, which has been used for different pathologies for approximately 400 years. This plant is rich in guanidine compounds that produce hypoglycemic effects in animals. In the early twentieth century, research showed that guanidines produce hypoglycemic effects in animals, but human use was avoided due its toxic effects [1].

The discovery of insulin and its subsequent extraction and purification from bovine pancreas decreased the interest in oral antidiabetic drugs until the mid-twentieth century. Jean Sterne, a French physician, was the first one that described the hypoglycemic effects of metformin after using it in diabetes patients [2–4]. Metformin was associated with lactic acidosis because it belongs to the same family that is phenformin, which prevented FDA approval of metformin as an oral hypoglycemic agent until 1995 [5]. Currently, metformin is one of the most produced oral antidiabetic drugs in the world and is considered the first-line treatment option managing type 2 diabetes* mellitus*. Recently, additional therapeutic actions have been described in other pathologies, such as polycystic ovary syndrome, obesity and hepatic steatosis, and potential uses in breast cancer [4, 6, 7].

A prospective follow-up of diabetic patients through the UKPDS (United Kingdom Prospective Study) indicated a decreased risk of death from all causes, including heart attack and diabetes-related deaths, in patients treated with metformin deaths compared with patients treated using other hypoglycemic agents. An associated decrease in stroke risk was reported, but the decrease was not statistically significant [8]. These observations are consistent with other reports that describe similar results. In 2003, Scarpello described positive effects, wherein, independent of glycemic control, mortality due to cardiovascular events and diabetes was reduced in patients taking metformin [9]. Roussel and Travert followed up 19,691 diabetic patients for one year. These authors observed lower mortality in a group of patients taking metformin [10].

Cheng et al. conducted a prospective study in Thailand for 4 years based on its electronic medical records. They followed up 14,856 patients and estimated a nearly 50% reduction in stroke episodes (adjusted hazard ratio 0.46, 95% CI: 0.424–0.518, *p* < 0.001) in patients with diabetes treated with metformin versus patients who did not use metformin [11]. The clinical benefit of metformin in diabetic patients for stroke prevention was described in a retrospective analysis by Mima et al. who reported a reduction in the severity of neurological symptoms [12].

Opposing results have also been reported for other pathologies of nervous system. In a case-control study, patients in the group treated with metformin alone versus other oral antidiabetic agents exhibited an increased risk of Alzheimer's disease (adjusted OR 1.71, 95% CI: 1.12–2.60 versus other oral agents such as sulphonylurea adjusted OR 1.01, 95% CI: 0.72–1.42) [13].

These epidemiological observations have increased interest in elucidating the mechanisms of action of metformin to explain these positive effects. In neuroscience, the search for a neuroprotective agent has been intense with several promising cellular models, although unsuccessful in its applicability in humans, partly due to the great distance separating these biological experiments with the human brain. Metformin is one of these molecules with neuroprotective potential that has been studied in various experimental models such as neuronal cultures and models of cerebral ischemia in rodents. These have allowed observation of their cellular effects at the neuronal, glial, and endothelial levels as well as cellular pathways such as apoptosis, neurogenesis, angiogenesis, and inflammation. This review focuses on the effect of metformin in brain ischemia in rodents and the molecular targets identified as part of the processes that explain the epidemiological phenomena discussed.

## 2. Pharmacological Aspects of Metformin

Metformin is a small, amphoteric, hydrophilic molecule with low lipid solubility and 12.4 pKa, which favors the acid (protonated) state in the intestine. Metformin requires an organic cationic transporter (OCT_1,2_) to enter the hepatocytes [14]. In humans, intestinal absorption yields a final bioavailability of approximately 50%. An inverse relationship between absorption and dose has been observed, which suggests a saturable process [15]. Metformin is not metabolized in the kidney, from which it is excreted without change and with a half-life of 4-5 hours. Compared with other biguanides, such as phenformin, metformin is less potent and does not produce its pharmacological effects in humans until it reaches 2700 mg/day. This low potency may explain its superior safety profile compared with other biguanides, especially its relationship with lactic acidosis [16]. The cationic transporter OCT_2_ is expressed in the kidney endothelium, which is involved in metformin elimination. This transporter has also been identified in neurons [17] and involves passage of metformin from systemic circulation to the neuronal interior. Toxicity was observed in rats at doses of metformin greater than 900 mg/kg/day delivered by gavage [18].

Metformin must cross the blood-brain barrier (BBB) to produce its neuroprotective actions. For this issue, observations in obese patients with and without diabetes treated with metformin show a decreased in food intake [14, 19]. Lv et al. assumed that these effects could be mediated by orexigenic and/or anorectic peptide expression or inhibition. Therefore, the authors evaluated metformin concentrations in cerebrospinal fluid (CSF) and in the hypothalamic region of obese diabetic male rats and observed concentrations at approximately four percent (4%) in the CSF compared with the plasma concentration [20]. Łabuzek et al. also demonstrated the presence of metformin in brains of Wistar rats treated with acute and chronic doses of 150 mg/kg metformin in a model of systemic inflammation induced by adding low doses of lipopolysaccharide (LPS) at 2 *μ*g/kg/day. Groups treated with metformin (acute and chronic) without LPS showed the highest concentration of metformin in the pineal gland and cerebellum and low concentrations in the striatum. In the LPS-treated groups, a higher metformin concentration was observed in the hypothalamus and striatum, suggesting that mild inflammatory conditions, which have been described for diabetes and atherosclerotic disease, facilitate metformin transport into the brain [15].

## 3. **Metformin** Action Mechanism

Although metformin has been approved in Europe since 1957 for treating type 2 diabetes* mellitus*, and several cellular pathways studies showed changes in the presence of metformin, the exact metformin action mechanism is unknown (Table 2). Its hypoglycemic action is due to reduced gluconeogenesis, increased insulin receptor sensitivity, especially in muscle cells, and decreased glucose uptake in the intestine [4, 20].

### 3.1. The Effects on AMPK

Zhou et al. (2001) determined that AMP-activated protein kinase (AMPK) is a cellular target of metformin by observing that AMPK activation was required for the metformin inhibitory effect on hepatocyte endogenous glucose production and for the decrease expression of the lipogenic gene SREBP-1. Zhou et al. found that AMPK activation could explain metformin's pleiotropic effects [21]. AMPK is a highly conserved serine/threonine kinase with three domains: *α*, *β*, and *γ*. The *α* subunit contains the catalytic site; two isoforms have been described: *α*_1_ and *α*_2_. The *γ* subunit comprises the regulatory sector that prevents permanent phosphorylation of a threonine (Thr172) in the *α* subunit [22]. AMPK monitors energy to protect cellular functions through sensing the AMP/ATP and ADP/ATP rates of change. AMPK is activated by increased cytoplasmic concentrations of AMP, which have been shown in nutrient deprivation, vigorous exercise, and ischemia. The physiological changes observed during AMPK activation induce a catabolic state and inhibit cellular processes that require high ATP consumption, such as protein, glucose, and lipid biosynthesis [23]. AMPK activation by both changes in the AMP/ATP intracellular rate and the AMPK upstream activator LKB1 leads to decreased gluconeogenic gene transcription.

### 3.2. The effects on Complex I of the Mitochondria Respiratory Chain

Foretz et al. showed that the hypoglycemic effects of metformin were maintained in AMPK and LKB1 knockout mice, which indicated that metformin produces such effect through an indirect mechanism [24]. Previously, El-Mir et al. observed less mitochondria oxygen consumption in the presence of biguanide, which suggests that this organelle, especially complex I of the mitochondrial respiratory chain, is the site of pharmacological action for metformin [25]. Interference with electron flow and a decrease in endogenous glucose production suggest that metformin-dependent cellular energy depletion leads to insufficient levels of ATP for hepatic gluconeogenesis [19]. Viollet et al. propose that inhibiting ATP production in mitochondria increases AMP levels with a consequent activation of AMPK. This AMPK activation decreases gluconeogenesis because an energy substrate for this metabolic process is not available [14]. At the neuronal level, the absence of enzymes in the glycolytic pathway decreases the neuronal ability to store nutrients. Intense and generalized AMPK activation was observed during cerebral ischemia in both ipsilateral and contralateral regions [23].

**Figure 1:**
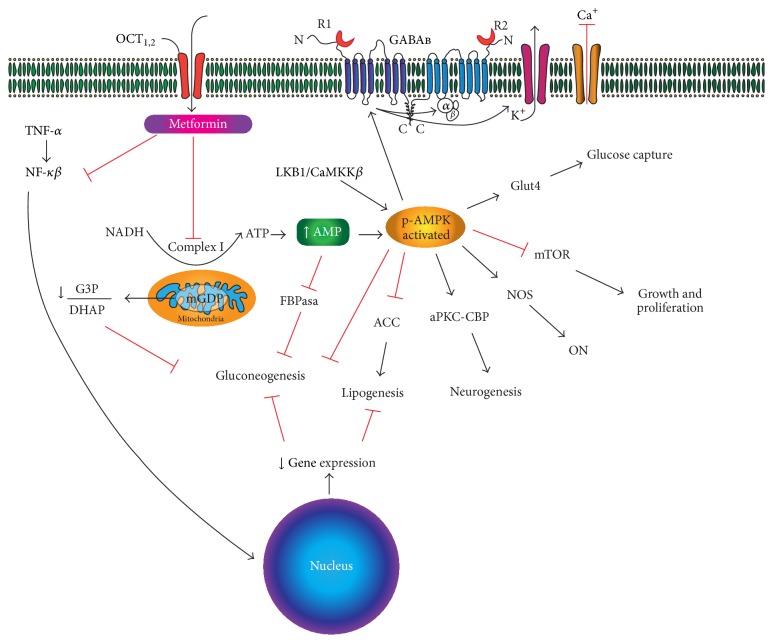
Different cellular pathways described for metformin function. Gluconeogenesis is inhibited due to changes in gene expression (such as for lipogenesis), FBPase inhibition, and changes in the ATP/AMP, G3P/DHAP, and lactate/pyruvate ratios. AMPK may exert different actions in neurons because they do not have glycolytic enzymes and, thus, a reduced ability to store nutrients and respond to energy deficit with a higher risk of lactic acidosis. The GABA_B_ receptor is involved in the effects of metformin through inducing hyperpolarization secondary to K^+^ release and inhibition of Ca^2+^ entry secondary to AMPK activation. OCT: organic cation (proton) transporter. G3P: glycerol-3-phosphate. mGDP: mitochondrial glycerophosphate dehydrogenase. DHAP: dihydroxyacetone phosphate. eNOS: endothelial nitric oxide synthase. ON: nitric oxide. aPKC: atypical protein kinase C. CBP: CREB binding protein. NF-KB: nuclear factor kappa beta. TNF-*α*: tumor necrosis factor-alpha. FBPasa: fructose 1,6 bisphosphatase. ACC: acetyl CoA carboxylase. CaMKK*β*: protein calmodulin-dependent kinase beta. mTOR: target of rapamycin in mammalian cells.

An increase in lactate and glycerol plasma levels has been observed in animals treated with metformin, but the increase cannot be attributed to reduced activity for the enzymes involved in pyruvate metabolism, such as pyruvate carboxylase, citrate synthase, and alanine aminotransferase. The increase in plasma lactate may indicate the cytosolic redox state by affecting the lactate/pyruvate rate. Madiraju et al. showed that acute and chronic metformin actions reduce endogenous glucose production by raising the cytosolic redox state and decreasing the mitochondrial redox state through noncompetitive inhibition of mitochondrial glycerophosphate dehydrogenase [26]. As a result, the balance between glycerophosphate/dihydroxyacetone phosphate, NADH/NAD^+^, and lactate/pyruvate in cytoplasm is affected, which decreases gluconeogenesis and releases excess lactate and glycerol to the plasma (Figure 1) [27].

### 3.3. Other Possible Targets of Metformin

Metformin interaction with metals has been described since the last century just after its synthesis and even before the description of its hypoglycemic effects [28]. Recently, a possible mechanism of action associated with copper ion binding has been described. Copper is essential for aerobic life given its presence as a catalytic cofactor for mitochondrial enzymes responsible for ATP synthesis [29, 30]. The available crystallographic and spectroscopic evidence that demonstrates the binding of metal ions to metformin contrasts with the lack of information about its affinity or its regulatory actions over certain proteins [19]. However, it is uncertain whether the effects of metformin on the mitochondrial complex I are related to its affinity for copper atoms that make part of metalloenzymes or cofactors in the respiratory chain. In relation to this, Logie et al. (2012) published a study on the properties of metformin relative to metal binding, particularly mitochondrial copper, and the effects of metformin relative to AMPK activation dependence in the presence of metals. The authors suggest that the cellular effects of metformin depend on its metal-binding properties [28].

## 4. **Studies on Metformin's Neuroprotective Effects in Rodent Animal Models**

The model of cerebral ischemia of the middle cerebral artery in rodents (MCAO) was initially proposed by Koizumi 30 years ago (with some later variations suggested by Longa et al.) and consists in the introduction of a monofilament (4-0 or 5-0 with a poly-L-lysine or silicone-rubber cover at the tip) by carotid internal artery to ascend it to the origin of the middle cerebral artery and producing a localized cerebral ischemia [31–33]. This model has been used to study different cellular pathways using metformin as described below.

### 4.1. AMPK Actions

Neurons are sensitive to energy deficits; thus, the high levels of AMPK expression observed in the central nervous system during ischemic events are unsurprising. However, the role of AMPK in neurons and its protective or harmful effects on brain ischemia remain controversial [34]. Harada et al. observed neuroprotective effects of intraperitoneal administration of metformin (250 mg/kg) through the peripheral activation of AMPK, after suppressing the glucose intolerance 24 hours after, reporting a less alteration in the mnemonic tests after three days of focal cerebral ischemia in mice [35]. In this study, Harada et al. also injected intraventricular metformin and observed an increase in the neuronal damage with neurological alterations, which suggests that central versus peripheral activation of AMPK may explain differences between neuroprotective and harmful metformin effects after ischemia.

Kuramoto et al. also described neuroprotective effects upon AMPK activation in a cerebral ischemia rodent model. These researchers observed enhanced activation of the metabotropic receptor GABA_B_ and its inhibitory actions through coupling to a postsynaptic G protein receptor (G_i_/G_o_) with neuronal hyperpolarization, which reduced secondary excitotoxicity during cerebral ischemia [36] (Figure 1). McCullough et al. described opposing results in 2005 upon observing neuroprotective effects using an inhibitor of AMPK referred to as compound C and knockout mice for AMPK*α*1 and AMPK*α*2 [37]. In this study, McCullough et al. were the first to report an association between the effects of activated AMPK and neuronal nitric oxide synthase (nNOS) in a cerebral ischemia mouse model (see below). During ischemic events, AMPK activation in neurons produces deleterious effects that are likely secondary to its reduced glycolytic capacity [19]. Therefore, prolonged production of ATP molecules through anaerobic glycolysis by astrocytes can lead to acidosis and inhibit lactate use by neurons as an energy substrate. Li et al. were more specific in describing the activity of each isoform, AMPK*α*1 and AMPK*α*2, to demonstrate that inhibition or acute inactivation of the *α*2 isoform is responsible for the neuroprotective effects in cerebral ischemia mouse models [38].

Later, Li et al. (2010) tested the neuroprotective actions of metformin using a mouse model of cerebral ischemia for 90 minutes (ischemia/reperfusion) and reported AMPK activation with acute metformin treatment and downregulation with chronic metformin treatment. The authors also reported an increase in infarct size in the groups that received acute doses of metformin (24 and 72 hours before the stroke) versus a decrease in infarct area with treatment for 3 weeks prior to ischemia [39]. No differences were observed in the cerebral infarction volume with the acute metformin treatment in AMPK*α*_2_ knockout mice versus the control group. The increase in infarct size from the acute ischemic events was not observed in neuronal nitric oxide synthase- (nNOS-) knockout mice. This effect led the authors to suppose that the neuroprotective effects were mediated by nitric oxide (see below). However, a metformin treatment for three weeks before ischemia decreased AMPK activation and the infarct size [39]. Based on these results and the assumption that less AMPK activation may yield neuroprotective effects, Mccullough and Benashski investigated ischemic preconditioning with 3 minutes of ischemia 72 hours before definitive MCAO, which showed neuroprotective effects in mice secondary to the AMPK inhibition and increased HSP70 expression [40].

### 4.2. Nitric Oxide Production and Oxidative Stress

After focal ischemia, events that cause damage or cell death involve a quick fall of adenosine triphosphate concentration (ATP), oxidative stress, changes in membrane potential with axonal depolarization, and massive entrance of sodium, chloride, and calcium ions and exchange of potassium ions [41]. The intracellular increase in calcium concentration promotes the production of nitric oxide, through the activation of constitutive synthases (endothelial and neuronal), which contributes to the initial damage. The generation of reactive oxygen species (ROS) became worse during reperfusion through the production of synthases involved in these processes [42]. Cerebral ischemia induces changes in the nitric oxide concentration in the brain 20 times the basal value, after middle cerebral artery occlusion for 30 minutes [43]. During ischemia, NO concentration decreases by the reduction of oxygen availability, so the constitutive endothelial isoforms, eNOS and nNOS, synthetize low levels of NO [44]. However, mRNA levels that encode nNOS increase in the neurons of the core and the penumbra zone after the cerebral ischemia [45, 46]. In the first hours after ischemia, high levels of NO are observed, mainly during reperfusion by overactivation of nNOS [47]. The inducible isoform of the nitric oxide synthase (iNOS) nondependent on calcium concentration is not expressed in nervous cells, but its activity increases mainly in glial cells, 12 hours after ischemia, and maintains high concentrations of ON in the brain, at least for 8 days especially in ischemic region [42, 48]. The transformation of nitric oxide into superoxides during reperfusion could be the cause of the additional damage to the nervous tissue, so the inhibition of the inducible nitric oxide (iNOS) may have neuroprotective effects by decreasing the nitrative stress [44, 49].

Therefore, the role of NO in the ischemic event may be dual due to its neuroprotective or neurotoxic effects [42, 43, 45, 48, 50]. NO, synthetized at low concentrations by eNOS, has local vasodilatory effects with good results in experiments that induce cellular damage after controlled ischemia [51]. These vasodilatory properties also have been described for nNOS when placed in the aortic endothelium [52]. In endothelial cells, a relation between cellular exposure to metformin and the activation of AMPK with secondary eNOS phosphorylation has been described in cardiac ischemic models and in muscle cells [53]. Although this results cannot be extrapolated to brain tissue, the supply of L-arginine (NO precursor) and the specific inhibitors of nNOS and iNOS in the murine cerebral ischemia model produce a reduction in the size of the infarct. In the case of eNOS, knockout mice for eNOS showed wide infarct areas [50]. During reperfusion, NO concentrations increased at the beginning due to the activation of nNOS and iNOS damaging neurons at the penumbra zone [42, 47, 54]. The effects of NO produced by the early activation of eNOS could be neuroprotective during the first 2 hours by promoting the collateral circulation, inhibiting platelet aggregation, and decreasing the leukocyte adhesion and by antiapoptotic effects [45, 48]. Hence, early eNOS activation could be a therapeutic target given biological chance of its neuroprotective effects. An initial approach with cellular studies in the endothelium showed, at this level, metformin granted the activation of eNOS and the bioactivity of NO secondary to the activation of AMPK in cell cultures of aortic mice endothelial cells [55]. At the neuron level, in poststroke mice, it has been noticed that the activation of AMPK depends on the production of NO by nNOS [39]. As we mentioned before, in rats subjected to ischemia with reperfusion, neuroprotective effects of metformin were observed during the chronic treatment before ischemia. These effects were not observed in knockout rats for nNOS [39]. These findings suggest that the selective inhibition of iNOS and activation of nNOS and eNOS may have neuroprotective effects.

### 4.3. Oxidative Stress

The excess NO production after cerebral ischemia and its deleterious effects on the activation of NOS and iNOS, as well as the continued neuronal death several hours and days after the stroke, suggest that molecules with antioxidant properties may have a neuroprotective effect. Thus, the nitrative stress described in the ischemia/reperfusion murine models suggests nitration increases of the p58 regulatory subunit of AKT (protein kinase B), favoring the activation of the apoptotic p38/MAPK kinase route. By the application of metformin, Correia and Carvalho observed a functional recovery of cerebral vessels in Goto-Kakizaki diabetic rats after focal cerebral ischemia by a reduction of nitrotyrosine in the endothelial cells of the microvasculature and an increase of AKT phosphorylation suggesting additional antioxidant effect [56]. Correia and Carvalho also evaluated the antioxidant action of metformin in Goto-Kakizaki rats by measuring levels of protein oxidation using peroxynitrite, glutathione, vitamin E, glutathione peroxidase, and superoxide dismutase enzymes. After comparing treatment groups, they reported a protective effect against oxidative stress through lower levels of lipid peroxidation markers as well as glutathione peroxidase and glutathione reductase activity [56].

Moustaf and Mohamed observed similar results as reported by Correia using an ischemia/reperfusion Wistar rat model by occluding both common carotid arteries for 30 minutes and then reperfusing for 60 minutes. The rats treated with metformin administered via a gavage tube (500 mg/kg/day) exhibited less oxidative stress with lower superoxide dismutase, glutathione peroxidase, and catalase activities than the sham operated group [57]. These results show that metformin may impact oxidative stress induced by cerebral ischemia and may impact antioxidant properties useful in controlling the side effects of ROS.

### 4.4. The Blood-Brain Barrier and Vascular Actions of Metformin

The blood-brain barrier (BBB) constituted by endothelial cells, perivascular cells (pericytes), and astrocytes feed endings performed a complex interphase between blood circulation and the brain parenchyma which keep the homeostasis of the central nervous system through the regulation of the ionic exchange, neurotransmitters, macromolecules, and nutrients [58, 59]. The breakdown of BBB during focal cerebral ischemia is associated with cerebral edema which favors the hemorrhagic transformation of ictus [60]. Given the diverse functions of the BBB and the few drugs with therapeutic actions at this level, Takata et al. observed metformin action dependent of the activation of AMPK in BBB functions by increasing transendothelial electrical resistance and decreasing sodium permeability in vitro using a culture of endothelial cells obtained from rat brains suggesting a strength of the endothelial cells tight junctions [61]. Farbood et al. evaluated the actions of metformin in the BBB in a global transitory ischemic model for 30 minutes, through the extravasation of Evans blue and the reactive hyperemia secondary to the ischemia. They observed that metformin decreases the BBB rupture and the neuronal excitability increases in the hippocampus [62]. Metformin actions and their relationship with AMPK activation at the blood-brain barrier have been studied. Liu et al. described the protective effects of metformin (200 mg/kg intraperitoneally) through altering the blood-brain barrier subsequent to MCAO for 90 minutes in mice by decreasing its permeability.

Additionally, the authors of this study evaluated the anti-inflammatory effects of cerebral ischemia followed by metformin, which is important considering the secondary inflammation that hypoxia triggers and neuronal death by necrosis in the affected brain parenchyma (or ischemic core). Previous studies show that metformin induces cellular change from an inflammatory to an anti-inflammatory phenotype. Consistent with these data, researchers have observed a decrease in expression of proinflammatory cytokines (IL-1*β*, IL-6, and TNF-*α*) and neutrophil infiltration subsequent to cerebral ischemia (the third day) through inhibiting expression of intercellular adhesion molecule (ICAM-1) secondary to AMPK activation [63]. Interestingly, unaltered occludin structure in the tight junctions area has been observed after metformin application as in the* zonula occludens* in the duodenal endothelium secondary to hepatic steatosis due to an elevated fructose diet [64].

These results show a contradiction in the neuroprotective effects of metformin since, at the level of BBB, the AMPK activation favors the tight junctions and reduces the efflux of fluid to the interstitial space; but at the neuronal level, the acute AMPK activation during stroke has shown negative effects in these cells. It is possible that not only temporary factors, like chronic versus acute treatment, but also the activation of different cellular pathways, for instance, the tyrosine-dependent phosphorylation, the age of the biomodels, or even the presence of diabetes, can be the cause of these paradoxical results.

In diabetic rats, neovascularization of the brain vessels with a major number of collaterals and an increase in the vascular tortuosity especially at the cerebral cortex have been observed. However, this increase in angiogenesis is not related to the proper maturation of the blood vessels wall because a decrease in the number of pericytes is observed around the endothelial cells and also an increased amount of nonperfused vessels [65]. Prakash et al. assessed the vascularization pattern in both hemispheres using diabetic rats undergoing cerebral ischemia for 90 minutes. They compared the effects of metformin administered orally 24 hours after ischemia at 300 mg/kg/day. They reported a decrease in the reparative neovascularization pattern in diabetic rats exposed to metformin after ischemia with a better functional outcome in the group with glycemic control [66]. The reduction of the osmotic changes secondary to the ischemic events, whether caused by damage to the BBB or by neuronal death, can cause distant effects due to the secondary edema in some cases. Control of edema and BBB damage may explain the positive results after metformin use in reducing the size of the infarct and on the improved motor performance during the postischemia period in murine stroke models.

### 4.5. Vascular Actions

The protective actions by metformin at the vascular level were assessed by Elgebaly et al. in a cerebral ischemia diabetic rat model. This model was exposed to minocycline and metformin for 4 weeks to determine their effects on vascular remodeling under such conditions, the role of matrix metalloproteinases (MMP) 2 and 9 in these vascular changes, and the risk of hemorrhagic transformation after MCAO ischemia. The authors observed an increase in the vascular remodeling markers indicating vessel tortuosity, number of medial (MCA) collaterals, number of anastomoses, diameter of the collateral arteries to the MCA, and activity of MMP-9 in diabetic rats. The rate of vascular remodeling was significantly reduced in the groups exposed to metformin, which also showed an association with reduced MMP-9 enzymatic activity, a smaller infarction area compared with controls, and a vasoprotective effect through preventing subsequent hemorrhagic transformation [67]. This “vascular antiremodeling” effect could explain the significant reduction of ECV events in diabetic patients treated with metformin, as seen in the diabetic patient follow-up study by Cheng et al. [11].

### 4.6. Neuronal Apoptosis Secondary to Cerebral Ischemia

One of the most interesting aspects of metformin is its antiproliferative action by inhibiting the mTOR complex in certain cancers. But the mechanism by which this effect may explain its neuroprotective action is contradictory [6, 14] since, in terms of neuroprotection, avoiding neuronal death by apoptosis inhibition may be beneficial. El-Mir et al. reported neuroprotective effects from metformin through preventing cell death in cortical primary neurons by inhibiting pore opening and mitochondrial permeability in the presence of neuronal death inducer (etoposide) [25]. The neuroprotective actions of metformin alone (or in combination with thymoquinone) were also described for a primary culture of cortical neurons exposed to ethanol (100 mM). The actions include less neuronal death, inhibition of impaired cytoplasmic calcium homeostasis, inhibition of an altered mitochondrial gradient, increased Bcl-2 (antiapoptotic) expression, and decreased Bax (proapoptotic) expression, which reduced the cytochrome c release and repressed caspase-9 [68]. Ashabi et al. used a global cerebral ischemia rat model (with 4 cerebral vessels occluded) and observed that preconditioning with metformin at 200 mg/kg/day by gavage for two weeks attenuated apoptosis of neurons located at CA1 in the hippocampus through measuring the effects on Bax/Bcl-2, caspase-3, and PARP levels. Further, metformin use favored mitochondrial biogenesis of certain proteins (PGC-1*α*, proliferator activator receptor gamma coactivator-1*α*). These effects were eliminated with compound C (selective and reversible AMPK inhibitor). The metformin doses were 100, 200, and 400 mg/kg/day orally; better neuroprotective results were observed for the 200 mg dose, which, based on the above-described pharmacokinetic properties, is the concentration that should exhibit better bioavailability (Table 1) [69].

### 4.7. Neurogenesis and Angiogenesis after Cerebral Ischemia

Neurogenesis is a characteristic of certain neuronal types that remain with mitotic activity throughout their life span. The generation of new neurons after an ischemic event may have neuroprotective effects, as long as order and stability are maintained to provide integration of new neuronal tissue and its functionality. Jin et al. found that, through the aPKC-CBP pathway, metformin promotes neurogenesis in the hippocampus of adult mice with improved performance in spatial memory testing [77].

The production of new blood vessels is a phenomenon that has been observed in studies with metformin as well. A rapid angiogenesis could be a valuable therapeutic tool but angiogenic therapies must have a time frame or window that allows the development of new blood vessels prior to the stroke. In accordance with this, Venna et al. observed neurological improvement and revascularization of ischemic tissue in mice after the administration of intraperitoneal metformin during 3 weeks prior to the stroke [72]. These angiogenic effects were dependent on AMPK*α*2. During the acute administration of metformin, angiogenesis can have neuroprotective effects after the stroke. Liu et al. observed that, after 14 days of metformin administration to ischemic mice, angiogenesis increased in the subventricular area, minimizing cerebral injury [74]. These effects were mediated by the activation of AMPK.

### 4.8. Inflammatory Pathway Actions

Metformin actions by suppressing the activation of inflammatory routes has been studied in different cell types like hepatocytes, adipocytes, and neurons [78, 80].

As mentioned previously, inflammation control secondary to ischemic events is a therapeutic target that has been studied for its potential neuroprotective effect. In 2003, Jung et al. used a AMP analogue AMPK-activator referred to as AICAR and observed proapoptotic effects in neuroblastoma cells associated with activation of this pathway [81]. Thereafter, several cellular targets have been described for AICAR, which may explain these contradictory results with the neuroprotective actions of metformin [38]. Moiseeva et al. previously described metformin actions in inhibiting a senescent phenotype through inhibiting NF-B*κ* translocation to the nucleus and repressing transcription of inflammatory cytokines genes [82].

Zhu et al. demonstrated that chronic preconditioning with metformin provides neuroprotection in permanent cerebral ischemia in rats by reducing the cerebral infarction area. The data showed improved neurological deficit through suppressing the inflammatory pathway mediated by nuclear factor kappa beta (NF-*κ*B) with reduced expression of proinflammatory cytokines as well as astrocytosis and secondary microgliosis during the first 24 hours of ischemia [71]. Ashabi et al. showed similar results when they analyzed anti-inflammatory and antioxidant actions after application of metformin in a global ischemic transitory model in rats (4 vessels). They determined reduced levels of TNF-*α*, NF-*κ*B, COX-2, Nrf2, catalase, and hemooxigenase-1 when compared with control group and sham rats. When an inhibitor of AMPK was used, the effect of metformin was counteracted [78].

## 5. Concluding Remarks

No currently available drug has a neuroprotective-approved indication for use in humans. To date, primary prevention is the best option to avoid complications associated with stroke. However, due to its high morbidity and mortality, the impaired quality of life of the surviving patients and their families, and the increase in life expectancy of humans, investigating drugs for neuroprotective effects is necessary for patients with a high stroke risk.

In recent years, metformin has garnered growing interest due to its likely neuroprotective actions and epidemiological observations on the mortality associated with its use in diabetes patients. Rodent models studies indicate that metformin produces favorable effects for preventing stroke and aftermath recovery actions that are independent of its hypoglycemic effects. Metformin effects at the vascular level have been described also in experiments with myocardial and endothelial cells. In all of them, it is suggested that the effect of metformin is via enhancing focal eNOS expression [74]. AMPK activation in hepatocytes and neurons involves an indirect mechanism relative to both its actions in glycemic control and its neuroprotective effects. Directly or indirectly, AMPK activation in the presence of metformin is associated with neuroprotective effects that involve the cellular pathways related to autophagia, microglial activation, a decrease in the movement of sodium through the BBB, angiogenesis, and neurogenesis.

In humans, this activation may produce endothelial protective effects that explain the lower cardiovascular risk observed in diabetic patients that use metformin [83] and some of these effects depend on the nitric oxide production by its endothelial isoform (eNOS).

Acute AMPK activation at the neuronal level produces harmful effects with an increased cerebral infarct area compared with chronic treatments for at least 2 to 3 weeks before ischemia in experimental biomodels [39]. Chronic treatment with metformin reduces AMPK activation and may produce similar effects as in studies on preconditioning before ischemia. However, activation of AMPK after cerebral ischemia for several days had demonstrated neuroprotective effects. The literature remains vague on the timing of beginning metformin for the acute condition; in the early hours of an ischemic event, metformin can produce deleterious effects, but long-term postinfarction administration may improve functional recovery in rodents [72, 77].

The different results for the cellular pathways studied in cerebral ischemia indicate that this molecule produces intrinsic neuroprotective factors because it affects the expression of neuroprotective genes, such as vascular endothelial growth factor (VEGF) [72]; affects oxidative stress by activating antioxidant enzymes, such as glutathione peroxidase [57, 84]; induces endogenous transcription factors through the aPKC-CBP pathway [70]; favors the M2 microglial phenotype associated with tissue repair [77]; inhibits cytochrome c release from the mitochondria; and induces neurogenesis in the hippocampus and subventricular zone [25, 72].

Biomodels tests in rodents are recommended to investigate potential drug candidates with potential neuroprotective effects because a standardized protocol is available, which facilitates correlations with brain cuts in specific areas. Unfortunately, many molecules have not proven equally effective when attempting to extrapolate the results in humans [85]. One reason could be the unidentified bias of using ischemia animal models without a clinical correspondence to humans, such as age, race, weight, sex, use of anesthetics for MCAO such as ketamine, and the time of ischemia. Comparing the results of neuronal cell culture versus in vivo results, the relationship between different types of cells in the nervous system, such as glia and neurons, cannot be simulated, and the drug concentrations used for irrigating a cell culture are much higher compared with bioavailability in a biomodel. Many studies focus on neuronal cell bodies in the cerebral cortex, the striatum, and/or hippocampus without considering axonal injury (white matter), which may also produce a major clinical impact. This consideration is relevant in humans, since the involvement of the internal capsule is very frequent and affects mainly the axons that go through.

The neuroprotective effects of metformin can be explained by its vascular actions (including its effects on the blood-brain barrier), neuronal actions, and glial actions based on the reported results. For example, its actions at the cerebral endothelium during ischemia indicate that it attenuates damage to blood-brain barrier through maintaining tight junctions, reducing sodium permeability, and reducing cerebral edema. Could this infarct area reduction effect with metformin in these biomodels be related to more than its neuronal actions? In addition, other pleiotropic metformin actions may also function in cerebral ischemia, such as suppressing mTOR, by which AMPK controls autophagy, cell growth, and secondary inflammation [63].

Thus, it should be considered that AMPK activation may produce temporary (acute and chronic) effects on cellular components of tissue (neurons, glia, and endothelium). This notion implies that neurons and glia activated pathways, gene expression, and cellular actions may differ, especially for the low glycolytic capacity of neurons and permanent postmitotic state [23]. Further, expression of the catalytic subunit of AMPK (*α*1 y *α*2) was described with a specific location among different cell types, which is important because the *α*2 subunit is involved in the neuroprotective effects. However, even though there is evidence that suggests that the effects of metformin could be mediated by its actions at a vascular level, the *α*1 subunit is mainly expressed in the endothelium, and the *α*2 subunit is mainly in the nucleus of neurons and the cytoplasmic region of astrocytes [86].

It seems paradoxical that the observed effects of metformin in certain cancer types (lung, prostate, ovarian, and melanoma), where it induces apoptosis of malignant cells, oppose the results described at the neuronal level [68, 87–89], which exhibit repression of this programmed cell death process and activation of neuronal signaling in stem cells. Metformin activation of the FOXO3-AMPK pathway that eliminates cells, which initiate gliomas by inducing differentiation into nontumor cells, has been described in the brain [90]. One explanation for these opposing effects of metformin in different tissues could be the postmitotic phase or cell cycle arrest in neurons, which maintain metabolic/enzymatic machinery that differs from and responds differently to the presence of biochemical signals, such as cyclins [91].

Metformin has pharmacological properties that should be more precisely defined in biomodels like rodents to determine the effectiveness of each clinical trial. To produce its hypoglycemic effects in humans, high doses of metformin ranging up to approximately 2,700 mg/day are typically required. This low pharmacological potency (compared with other oral antidiabetics) may impact research results in rodents, especially given the absence of a determined dose or a threshold where neuroprotective effects are observed. The doses used in rats and mice range between 10 mg/kg/day and 500 mg/kg/day with different administration routes (gavage and intraperitoneal), which can lead to significant changes in bioavailability of the molecule because, given its low absorption in the intestine and the high dose required to produce cellular effects, studies where metformin was administered by gavage can produce different results compared with intraperitoneal injection. Studies of these possible differences in bioavailability based on the administration route for metformin are still necessary. It is interesting, however, that the dosage used in humans of approximately 30 mg/Kg/day, when given intraperitoneally to rats 7 days before the stroke, does not have the neuroprotective effect [79].

Finally, other available oral antidiabetic agents, such as sulfonylureas and inhibitors of dipeptidyl peptidase-4 (DPP-4), show similar actions to metformin, which are outlined in this review as candidates due to antiapoptotic neuroprotective, anti-inflammatory, and antioxidant actions and attenuated microglial reactivity [92, 93].

## Figures and Tables

**Table 1:** Different cellular pathways studied on cultured neurons using metformin in vitro/in vivo model.

Biomodel	Culture	Effects	Postulated mechanism	Doses	Ref
Rat	Culture of primary neurons of day 17	Antiapoptotic	Pore opening inhibits mitochondrial permeability transition	100 *μ*g for 24 hours	[25]

Mice	Immunostaining hippocampal sections	Neurogenesis	Metformin activates the aPKC-CBP pathway in neurons and induces neurogenesis of human neurons in culture. In adult mice, it induces neurogenesis in the hippocampus and olfactory bulb. Spatial learning tests improved in mice	Daily injected metformin (200 mg/kg) and BrdU for 3 days, and then only metformin for 9 days	[70]

Rat	Culture of cortical neurons from embryos obtained on gestation day 17,5	Antiapoptotic	Decreases neuronal death, inhibits apoptosis activation, maintains the mitochondrial gradient, inhibits cytochrome c release, and regulates internal calcium homeostasis	Culture at a high concentration of ethanol and groups received metformin (100 mM) and/or thymoquinone	[68]

Rat	Primary cultures of rat brain endothelial cells	Maintained integrity of BBB	Decreases sodium pass and maintains transmembrane electric gradient	Metformin dissolved 1 mM	[61]

**Table 2:** Different in vivo studies using metformin in a model of focal or global ischemia with or without reperfusion. Most of the studies show neuroprotective effects. PO: oral, IP: peritoneal injection, ICV: intraventricular, and IPC: ischemic preconditioning.

Biomodel	Strain	Ischemic focal or global	Time of ischemic/reperfusion	Neuroprotection	Postulated mechanism	Doses and route of administration*✪*	Metformin treatment time	Ref.
Rat	Wistar	Global	30 min/1 h	Yes	Reduction superoxide dismutase activity and glutathione peroxidase	500 mg/kg PO	One week before stroke	[57]

Rat	Sprague-Dawley	Focal	Not reperfusion	Yes	Suppression of the NF-*κ*B inflammatory pathway	50 mg/kg IP	3 weeks before stroke	[71]

Mice	C57BL/6N	Focal	60 min/72 h	Yes	Angiogenesis and improve cerebral dopaminergic tone	0,2 mL/kg IP o 50 mg/kg/day IP	50 mg/kg/day beginning 24 h after stroke for 3 weeks	[72]

Rat	Sprague-Dawley	Focal	Not reperfusion	Yes	Induces autophagy	10 mg/kg/IP	Single dose 24 h before stroke	[73]

Mice	CD-1	Focal	90 min/14 days	Yes	Promotes neurogenesis and angiogenesis in subventricular zone	200 mg/kg IP	For 14 days after stroke	[74]

Rat	Goto-Kakizaki	Focal	90 min/14 days	Yes	Reduce levels of nitrotyrosine	300 mg/kg/day PO	For 14 days after stroke	[75]

Mice	C57BL/6N	Focal	3 min (IPC) at 72 hours 90 min/24 h	No	AMPK activation antagonizes neuroprotective effect of ischemic preconditioning	100 mg/kg IP	Single doses after stroke	[76]

Mice	CD-1	Focal	60 min/14 days	Yes	Favors change in phenotype M2 microglia/astrocyte	50 mg/kg/day IP	Beginning 24 h after stroke	[77]

Mice	C57BL/6	Focal	90 min/24 y 72 hours	No: acute treatment Yes: chronic treatment	In chronic treatment, less activation of AMPK	Acute: 50 a 100 mg/kg/día IP Chronic: 50 a 100 mg/k/day	Acute: 24 h before Chronic: 3 weeks before	[39]

Rat	Wistar	Global	30 min/72 h	Yes	AMPK activation inhibits apoptosis in hippocampal neurons	200 mg/kg/day PO	2 weeks before ischemia	[69]

Rat	Goto-Kakizaki	Focal	90 min/21 h	Yes	Reduced vascular remodeling and severity of hemorrhagic transformation in diabetes	300 mg/kg/day PO	Starting with the onset of diabetes	[67]

Rat	Wistar	Global	30 min/72 h	Yes	Attenuates cellular levels of NF-kB and increased levels of Nrf2 in hippocampus	200 mg/kg/day PO	2 weeks before ischemia	[78]

Rat	Wistar	Global	30 min/72 h	Yes	Decreases reactive hyperemia and permeability of BBB in ischemic rats	200 mg/kg/day PO	2 weeks before ischemia	[62]

Mice	ddY	Focal	60 min/72 h	No: acute injection intraventricular Yes: chronic, IP	Central versus peripheral activation of AMPK	250 mg/kg/IP three times or 25,100 *μ*g ICV three times for day	After stroke until death	[35]

Rat	Goto-Kakizaki	Focal	90 min/14 days	Yes	Improved vascularization in diabetic group, achieve euglycemia	300 mg/kg/day PO	After stroke until death	[66]

Mice	C57BL/6	Focal	60 min/24 h	Yes	In seven-day pretreatment, less activation of AMPK	10 mg/kg/day IP	After stroke until death	[79]
